# Hereditary Angioedema Prophylaxis Therapy: Berotralstat and Lanadelumab Safety Profile

**DOI:** 10.3390/medicina61111897

**Published:** 2025-10-23

**Authors:** Mattia Donadoni, Leyla La Cava, Emanuele Bizzi, Valentina Popescu Janu, Alessia Meschia, Federica Cirigliano, Chiara Cogliati, Antonio Gidaro

**Affiliations:** 1 Internal Medicine, L. Sacco Hospital, ASST Fbf-Sacco, 20146 Milan, Italy; 2 Fatebenefratelli Hospital, ASST Fbf-Sacco, 20146 Milan, Italy; 3 Department of Biomedical and Clinical Sciences, Università degli Studi di Milano, 20146 Milan, Italy

**Keywords:** hereditary angioedema (HAE), C1 inhibitor deficiency (C1-INH), long-term prophylaxis (LTP), berotralstat, lanadelumab, adverse drug reactions (ADRs), adverse drug reactions (ADRs)

## Abstract

*Background and Objectives*: Hereditary angioedema caused by C1 inhibitor deficiency (HAE-C1-INH) is a rare genetic condition characterized by recurrent, potentially life-threatening episodes of angioedema. Long-term prophylaxis (LTP) is essential for decreasing the frequency and severity of attacks. This study aims to compare the safety profiles of two first-line LTP therapies, both of which inhibit kallikrein: berotralstat (oral) and lanadelumab (subcutaneous), using data from the WHO’s VigiBase pharmacovigilance database. *Materials and Methods*: The study employed a retrospective quantitative design, utilizing the World Health Organization’s pharmacovigilance database, VigiAccess, which contains individual case safety reports of adverse drug reactions (ADRs) to identify cases of ADRs associated with HAE-C1-INH long-term prophylaxis. *Results*: A total of 644 reports for berotralstat and 3432 reports for lanadelumab were analyzed. Berotralstat was mainly associated with gastrointestinal adverse events (47.9%), while lanadelumab was linked to injection site reactions (45.9%), infections (23.3%), musculoskeletal and connective tissue disorders (10%), immune system disorders (5.3%), vascular disorders (4.7%), and metabolic issues (3.9%). Female patients were more frequently affected in both groups. Statistically significant differences were observed, reflecting the differences in administration methods and pharmacological profiles between the two drugs. Limitations include the self-reported nature of the data and the absence of detailed clinical information. *Conclusions*: The results confirmed the literature’s data on the gastrointestinal adverse effects of berotralstat, as well as site reactions and infections associated with lanadelumab. Notably, musculoskeletal and connective tissue disorders, immune system disorders, vascular disorders, and metabolic issues occurred more frequently in patients using lanadelumab.

## 1. Introduction

Hereditary angioedema due to C1 inhibitor deficiency (HAE-C1INH) is a rare autosomal dominant disorder caused by either C1 inhibitor deficiency (type I) or dysfunction (type II). It results in dysregulated plasma kallikrein activity, increased bradykinin production, and unpredictable, potentially life-threatening recurrent angioedema attacks [1]. HAE-C1INH is characterized by sudden, localized, and transient swelling of the deeper layers of the skin or mucous membranes, often affecting the face, lips, tongue, extremities, and genitalia. It may involve the upper airway, leading to potentially life-threatening laryngeal edema, or the gastrointestinal tract, causing abdominal pain, nausea, or vomiting. The swelling is usually non-pitting and non-pruritic, and can last from several hours to a few days.

Treatment guidelines recommend that long-term prophylaxis (LTP) be considered for every patient with HAE-C1INH to reduce the frequency and severity of attacks, and the decision to use LTP should be tailored to the individual’s specific needs [2]. Historically, oral prophylactic options included attenuated androgens and antifibrinolytics. However, while antifibrinolytics are not effective, oral attenuated androgens have many side effects that limit their tolerability, especially for women and children [3,4].

Currently, the first-line LTPs approved for patients with HAE-C1INH are plasma-derived C1-INH concentrate (pdC1-INH), lanadelumab, and berotralstat (Table 1). pdC1-INH concentrates can be given subcutaneously or intravenously. This LTP aims to replace the missing protein without causing significant adverse drug reactions (ADRs). However, local adverse events such as injection site pain, hematoma, bleeding, induration, and bruising may occur, especially in older HAE-C1INH patients [5]. Additionally, there is a substantial treatment burden due to the administration interval, which is every 3–4 days for both available formulations. The other two drugs, lanadelumab and berotralstat, work by inhibiting kallikrein activity.

Lanadelumab is a recombinant, fully human immunoglobulin G monoclonal antibody, administered subcutaneously every 2–4 weeks based on each patient’s disease control. Berotralstat is a small molecule that is administered orally in the form of a once-daily tablet. This study aimed to evaluate the demographic distribution of adverse drug reactions (ADRs) for berotralstat and lanadelumab. Additionally, we assessed whether the distribution of side effects was similar for both drugs, which share the same mechanism of action: inhibiting plasma kallikrein.

## 2. Materials and Methods

The study used a retrospective quantitative design, utilizing the World Health Organization’s pharmacovigilance database of individual case safety reports of ADRs, VigiAccess (https://www.vigiaccess.org/), to identify cases of ADRs associated with HAE-C1-INH long-term prophylaxis.

VigiBase is the WHO’s global database of adverse event reports for medicines and vaccines. It is the largest database of its kind worldwide, with about 40 million reports of suspected drug ADRs, and it is continuously updated with new reports. All cases in VigiBase are self-reported using MedDRA terms. Suspected adverse reactions are reported by both the healthcare provider and the patient to the WHO program for International Drug Monitoring. The program anonymizes the report and forwards it to the Uppsala Monitoring Centre, which then uploads it into VigiBase. Additional clinical details, such as diagnostic criteria and toxicity management, are not included. Each anonymous report may contain more than one ADR. Data on the ADRs of berotralstat and lanadelumab were collected on 6 July 2025.

The relative incidence of ADRs for the two drugs was analyzed to assess whether their safety profiles were similar. Only adverse reactions with an incidence greater than 3% in at least one of the drugs were considered. Fisher’s test was used to determine if there was a statistical difference in the relative incidence of ADRs; a *p*-value of <0.05 was deemed statistically significant. Adverse reaction data for berotralstat have been collected since 2021, while data collection for lanadelumab started in 2015.

## 3. Results

We identified 644 reports related to berotralstat, totaling 1051 adverse events, and 3432 reports regarding lanadelumab, which resulted in 8572 adverse events. Regarding berotralstat, reports were almost evenly distributed between Europe (305) and the Americas (337). In contrast, lanadelumab reports were significantly higher in America than in Europe (2744 vs. 660 reports).

**Figure 1:**
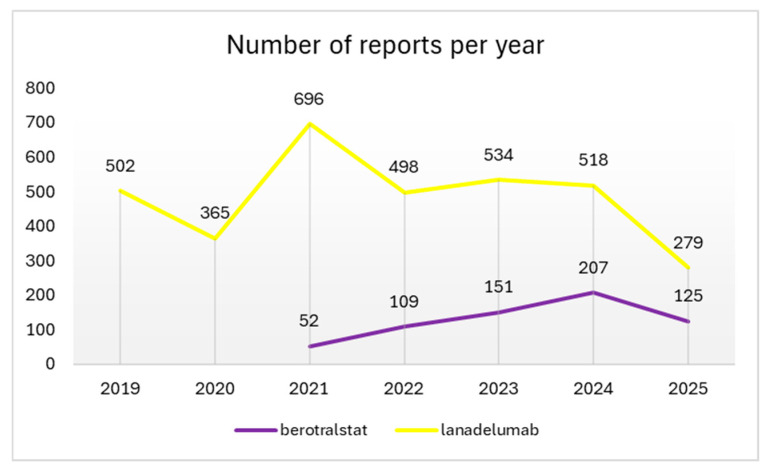
Reports of ADRs per year.

Over the years, the number of reports related to berotralstat has shown an increasing trend, rising from 52 in 2021 to 207 in 2024 and 125 in the first 6 months of 2025. In contrast, reports concerning lanadelumab have exhibited significant growth since 2019, maintaining a consistent annual volume except for a notable peak in 2021, when the number of reports nearly doubled compared to the preceding and subsequent years (Figure 1).

Considering the period during which both drugs have been available (starting from 2021, when berotralstat was released), the total number of adverse event reports for lanadelumab surpassed those for berotralstat by 1881 cases, with 2525 reports linked to lanadelumab and 644 to berotralstat. When examining the demographic distribution of events, female patients were predominantly represented in reports for both drugs, with a notably higher occurrence in the lanadelumab group (Table 2).

The most reported adverse reactions associated with berotralstat were gastrointestinal disorders, representing 47% of all reports and 29% of all adverse events. In contrast, for lanadelumab, the most common adverse reactions were related to complications from subcutaneous injections, making up 45.9% of total reports and 18% of total adverse events (Table 2). Berotralstat was more frequently linked to adverse gastrointestinal reactions (berotralstat, 47.8% vs. lanadelumab, 13.8%, *p*-value < 0.001). Even when analyzing specific subgroups such as diarrhea, abdominal pain, nausea, and dyspepsia, prevalence remained higher in the berotralstat group (Table 3).

Conversely, lanadelumab was more frequently linked with general disorders and conditions at the administration site (berotralstat 31% vs. lanadelumab 46%, *p*-value < 0.001), infections (berotralstat 8% vs. lanadelumab 23.3%, *p*-value < 0.001), musculoskeletal and connective tissue disorders (berotralstat 4.6% vs. lanadelumab 10%, *p*-value < 0.001), immunological disorders (berotralstat 1.4% vs. lanadelumab 5.3%, *p*-value < 0.001), and vascular disorders (berotralstat 2.6% vs. lanadelumab 4.7%), as well as issues related to metabolism (berotralstat 1.3% vs. lanadelumab 3.9%, *p*-value < 0.001) (Table 3).

## 4. Discussion

To date, no precise data are available in the literature regarding the relative prevalence of lanadelumab versus berotralstat use for HAE long-term prophylaxis. Although lanadelumab seems to be more commonly used as LTP than berotralstat across various studies, precise prevalence data are currently lacking [6,7,8].

Real-world evidence and observational studies indicate that lanadelumab was initially prescribed more often, especially for patients who preferred subcutaneous administration or had previous experience with injectable therapies [9]. However, berotralstat has gained popularity due to its oral route, which appeals particularly to patients seeking to avoid injections or facing difficulties with parenteral treatments. Recent data indicate that a substantial number of patients who initially started with lanadelumab later switched to berotralstat, primarily due to personal preferences and convenience factors [10].

Considering its earlier availability and broader initial adoption, it is not surprising that our study observed a higher number of adverse events for lanadelumab.

Our analysis showed a significant difference in report counts between the two drugs, with 644 reports of ADRs for berotralstat compared to 3432 for lanadelumab. The number of AE reports does not follow a linear pattern based on the usage prevalence of both drugs. Nonetheless, during the period when both drugs were available on the market simultaneously, lanadelumab-related reports exceeded those for berotralstat by 1881 cases.

The incidence of ADRs associated with berotralstat appears to be similar in Europe and America. However, lanadelumab is more commonly used in America, which might explain the higher number of AEs reported there [7]. Although berotralstat has gained approval from the U.S. Food and Drug Administration (FDA), the latest guidelines from the United States Hereditary Angioedema Association Medical Advisory Board recommend lanadelumab as the first-line treatment for LTP [11,12]. This difference is also likely due to berotralstat entering the market later, as indicated by the gradual increase in adverse event reports over time.

When evaluating sex-related differences, the prevalence of adverse events (AEs) seems higher in females for both berotralstat and lanadelumab. Hereditary angioedema generally shows a greater clinical burden in women, who tend to be more symptomatic than men [13]. This apparent female predominance may be partly due to increased symptom severity in women, which leads to earlier diagnosis and treatment initiation [14].

Women often report more frequent and severe angioedema attacks compared to men, leading to higher use of long-term prophylaxis (LTP) therapies and, as a result, greater exposure to potential AEs. In fact, increased estrogen levels, such as those seen during pregnancy or with oral contraceptive use, are well-known triggers for HAE exacerbations. A growing body of evidence indicates a link between high estrogen states and both increased frequency and severity of angioedema attacks in patients with HAE [15]. Estrogenic hormones can influence the expression of proteins involved in the kallikrein–kinin system, ultimately boosting the activity of the contact system. Consequently, female patients often experience a more severe disease phenotype. Puberty is a key period during which disease severity usually worsens in females, though an increased attack frequency has also been observed in males [16].

Although most reports did not specify patient age, the available data indicate a higher occurrence of adverse events (AEs) among individuals aged 18–44 years for berotralstat (15% of reports) and among both the 18–44 and 45–64 age groups for lanadelumab (each representing 24% of reports). This age distribution aligns with existing literature, which reflects the typical age at which lanadelumab therapy is initiated [9]. This may relate to the usual age of disease onset and, consequently, the start of LTP therapy. Additionally, the availability of these two drugs in the pediatric population (under twelve years) is recent, and their use in this age group is expected to increase soon.

Compared to lanadelumab, which requires subcutaneous injection, berotralstat offers the benefit of oral administration. Consistent with existing literature, our analysis confirmed that gastrointestinal adverse events were the most common in the berotralstat group. In our study, gastrointestinal adverse effects accounted for 47% of total reports, aligning with data reported by Zuraw et al. [7] and Farkas et al. [3], where combined gastrointestinal AEs occurred in 42% and 44% of the study population, respectively. Specifically, any significant gastrointestinal adverse effects (diarrhea, abdominal pain, nausea, and dyspepsia) were statistically more common in the berotralstat reports. Typically, gastrointestinal adverse effects occur within the first month of treatment and are mild and transient. Importantly, these symptoms tend to lessen with continued treatment and often resolve without the need for additional medication [17,18]. The role of microbiota in angioedema remains a topic of debate, as changes in gut microbiota have been observed in patients with recent episodes of HAE [19]. However, the relationship between LTP and microbiota requires more solid evidence, and further studies are needed to clarify their interaction.

On the other hand, the most frequently reported adverse drug reactions in patients treated with lanadelumab were injection-site related issues, such as pain or local erythema, as well as fatigue. These data were categorized under ‘General disorders and administration site conditions’, so it is not unexpected that injection-related effects are more prevalent among lanadelumab users, considering that berotralstat is orally administered (berotralstat 31% vs. lanadelumab 46%, *p*-value < 0.001). The six reports of injection site pain and erythema among berotralstat users must be considered an error. Injection-site related effects accounted for nearly half (45.9%) of all reports observed with lanadelumab. Injection complications are the main adverse effects associated with lanadelumab [20,21]. This data aligns perfectly with the findings described by Banjery et al. [1], which reported a prevalence of 43% for injection site pain, and is consistent with data reported by the US Food and Drug Administration (45–57%) [22].

Notably, lanadelumab was associated with immune system disorders (5.3% of reports), particularly drug hypersensitivity symptoms such as flushing, erythema, and pruritus, which are common side effects of monoclonal antibodies [23]. In the case of lanadelumab, the incidence seems higher than what Banerji et al. [1] reported, who documented only one case of drug hypersensitivity in their study, and it is also higher than the rate described by the U.S. FDA, which considers hypersensitivity a rare adverse event (about 1%) [22].

The link between lanadelumab and upper respiratory tract infections is well-supported in the literature. Notably, in the study by Banerji et al. [1], nearly one in four patients treated with lanadelumab experienced viral upper respiratory infections, especially during the loading dose administered every two weeks. In our analysis, a statistically significant link was found between lanadelumab and the occurrence of COVID-19 infection and pneumonia.

Lanadelumab showed a statistically significant link with infections (23.3% of all reports), even when COVID-19-related cases were excluded from the analysis. However, these results should be interpreted with caution, as a clear causal relationship cannot be confirmed. The increased reporting of COVID-19 and pneumonia in patients treated with lanadelumab likely reflects its broader use during the COVID-19 pandemic (2020–2022), which increased the likelihood of such events being recorded. Additionally, as Senter et al. [24] support, COVID-19 could worsen existing HAE or trigger the development of HAE in asymptomatic carriers. Moreover, the type of pneumonia (bacterial versus viral) was not specified, which may have led to misclassification of COVID-19-related symptoms and contributed to an overestimation of infection-related adverse drug reactions.

The higher prevalence of neoplasms among lanadelumab users may be attributed to the drug’s earlier market entry, which allows for a more extended follow-up period during which patients may develop neoplasms [25]. Additionally, immunosuppression is a well-known risk factor for the development of cancer. Therefore, we cannot rule out the potential influence of the loading dose of 300 mg administered every two weeks.

Lanadelumab was more often linked to a broad range of musculoskeletal and connective tissue disorders. However, when individual subcategories, such as myalgia, arthralgia, and back pain, were examined separately, none of the associations were statistically significant. Still, further research is needed to better understand the true incidence of these adverse events, especially since myalgia has already been reported as an adverse effect for other monoclonal antibodies, such as tezepelumab [26].

Additionally, lanadelumab showed a signal of association with changes in body weight, including both gain and loss, representing a new finding for this drug [20]. However, there is currently no clear explanation for this link, and more research is needed to understand its possible mechanisms and clinical significance.

Finally, lanadelumab was statistically linked to vascular disorders. Among the evaluated subcategories, only hemorrhage remained statistically significant; however, due to the limited sample size and the complete absence of such events in the berotralstat group, a definitive link cannot be confirmed. Additionally, although prolongation of activated partial thromboplastin time (aPTT) was seen in lanadelumab recipients, it was not associated with clinically apparent bleeding adverse events [22].

### Limits

All cases retrieved from VigiBase are self-reported and classified according to the MedDRA system, which limits the scope of our study. Specifically, detailed clinical data such as diagnostic criteria, severity grading, and toxicity management are not available. Additionally, the classification of adverse drug reactions (ADRs) is inconsistent, as similar or synonymous terms may be reported separately instead of being grouped under a standard category. This can lead to fragmentation and an underestimation of specific ADR patterns. Furthermore, the lack of information on the exact number of patients treated with lanadelumab or berotralstat limits a reliable comparison of the frequency of each adverse effect between the two treatments.

Moreover, the VigiAccess site provides all the ADRs together; for this reason, we cannot choose to evaluate the same number of ADRs for the two drugs.

## 5. Conclusions

Results confirmed the literature’s data on the gastrointestinal adverse effects of berotralstat, as well as site reactions and infections linked to lanadelumab. Notably, musculoskeletal and connective tissue disorders, immune system disorders, vascular disorders, and metabolic issues occurred more frequently in patients using lanadelumab.

## Figures and Tables

**Table 1:** Long-term prophylaxis is recognized as a therapeutic option for HAE. STP: short-term prophylaxis; LTP: long-term prophylaxis; iv: intravenous; sc: subcutaneous.

Drug Name (Trademark)	Indications	Dosage	Mechanism of Action
Plasma-derived C1 inhibitor (Cynrize)	Acute attack, STP, LTP All age groups	1000 IU iv every 3–4 days	Inhibits factor XIIa and activated plasma kallicrein
Plasma-derived C1 inhibitor (Haegarda, Berinert sc, CSL Behring)	LTP	60 IU/kg sc every 3–4 days	Inhibits factor XIIa and activated plasma kallicrein
Lanadelumab (Takhzyro, Takeda)	LTP	300 mg sc every 2 or 4 weeks	Inhibits activated plasma kallicrein
Berotralstat (Orladeyo, BioCryst)	LTP	150 mg daily orally	Inhibits activated plasma kallicrein

**Table 2:** Reports distribution per sex and age.

	Berotralstat (%)	Lanadelumab (%)
**Patient sex distribution**		
Female	238 (37%)	2433 (71%)
Male	128 (20%)	790 (23%)
Unknown	278 (43%)	209 (6%)
**Age group distribution**		
2–11 years	0 (0%)	21 (0.6%)
12–17 years	24 (4%)	91 (3%)
18–44 years	97 (15%)	817 (24%)
45–64 years	78 (12%)	821 (24%)
65–74 years	36 (6%)	199 (6%)
≥75 years	9 (1%)	98 (3%)
Unknown	400 (62%)	1385 (40%)

**Table 3:** The relative incidence of ADRs between berotralstat and lanadelumab. In bold, statistically significant results. Unk: unknown.

Adverse Events	Berotralstat (%)	Lanadelumab (%)	*p* -Value
**Cardiac disorders**	22 (3.4)	136 (3.9)	0.57
**Gastrointestinal disorders**	**308 (47.9)**	475 (13.3)	**0.0001**
Diarrhea	94	71	**0.0001**
Abdominal pain	66	44	**0.0001**
Nausea	52	94	**0.0001**
Dyspepsia	39	9	**0.0001**
**General disorders and administration site conditions**	200 (31)	**1575 (45.9)**	**0.0001**
Injection site pain	3	333	**0.0001**
Injection site erythema	3	114	**0.0001**
Fatigue	38	107	**0.0001**
**Immune system disorders**	9 (1.4)	**183 (5.3)**	**0.0001**
Drug hypersensitivity	4	99	**0.0002**
Anaphylactoid reaction	2	23	0.411
**Infections**	52 (8)	**801 (23.3)**	**0.0001**
COVID-19	16	237	**0.0001**
Influenza	5	73	**0.018**
Viral gastroenteritis	4	22	1
Pneumonia	0	105	**0.0001**
**Injury**	65 (10)	**1384 (40.3)**	**0.0001**
Product dose omission issue	14	600	**0.0001**
Inappropriate schedule of product administration	8	371	**0.0001**
Exposure during pregnancy	6	44	0.1633
**Investigations**	50 (7.7)	**683 (19.9)**	**0.0001**
Weight decreased	8	358	**0.0001**
Weight increased	15	362	**0.0001**
Blood pressure increased	3	39	0.139
**Metabolism**	10 (1.5)	**135 (3.9)**	**0.0016**
Decreased appetite	5	20	0.579
Dehydration	1	30	0.0105
Weight fluctuation	0	23	0.0626
**Musculoskeletal and connective tissue disorders**	30 (4.6)	**345 (10)**	**0.0001**
Myalgia	4	54	0.0684
Arthralgia	5	57	0.112
Back pain	11	44	0.3561
**Neoplasms**	13 (2)	**133 (3.8)**	**0.0202**
**Nervous system disorders**	111 (17)	600 (17,4)	0.91
**Product issues**	9 (1.4)	**268 (7.8)**	**0.0001**
Product available issue	2	102	**0.0003**
Product distribution issue	1	71	**0.0076**
**Respiratory, thoracic, and mediastinal disorders**	36 (5.6)	**319 (9.2)**	**0.0017**
**Skin and subcutaneous tissue disorders**	52 (8)	**380 (11)**	**0.0253**
**Social circumstances**	3 (0.4)	**221 (6.4)**	**0.0001**
Insurance issue	2	168	**0.0001**
Inability to afford medication	unk.	19	0.0589
**Surgical and medical procedures**	11 (1.7)	**230 (6.7)**	**0.0001**
Hospitalization	1	56	**0.0014**
Therapy interrupted	3	36	0.191
Surgery	17	162	0.1595
**Vascular disorders**	17 (2.6)	**162 (4.7)**	**0.0158**
Thrombosis	2	37	0.0759
Hemorrhage	0	23	**0.0391**

## Data Availability

Data are public and can be downloaded on VigiAccess (https://www.vigiaccess.org/).

## Abbreviations

The following abbreviations are used in this manuscript:

HAE-C1INH	Hereditary angioedema due to C1 inhibitor deficiency
LTP	Long-term prophylaxis
ADRs	Adverse drug reactions
pdC1-INH	Plasma-derived C1-INH concentrate
AEs	Adverse events
